# Anti-Remodeling Cardiac Therapy in Patients With Duchenne Muscular Dystrophy, Meta-Analysis Study

**DOI:** 10.3389/fphar.2021.769896

**Published:** 2022-01-20

**Authors:** Bruria Hirsh Raccah, Bar Biton, Offer Amir, Israel Gotsman, Dean Nahman, Ilan Matok

**Affiliations:** ^1^ Division of Clinical Pharmacy, Faculty of Medicine, School of Pharmacy, Institute for Drug Research, the Hebrew University of Jerusalem, Jerusalem, Israel; ^2^ Heart Institute, Hadassah Medical Center, Faculty of Medicine, Hebrew University, Jerusalem, Israel; ^3^ The Azrieli Faculty of Medicine in the Galilee, Bar-Ilan University, Safed, Israel

**Keywords:** heart failiure, ejection fraction, mortality, heart rate, BNP (B type natriuretic peptide)

## Abstract

**Background:** Almost all Duchenne muscular dystrophy (DMD) patients that reach their 30s present cardiomyopathy. As a result, this population remains under-treated. There is no sufficient proof of the efficacy of anti-remodeling cardiac therapy for DMD cardiomyopathy (DMDCM). We aim to assess the efficacy of anti-remodeling cardiac therapy for DMDCM by using meta-analysis.

**Methods:** PubMed (MEDLINE), Embase, and Cochrane library were searched through January 2021. Randomized control trials, case-control studies, and observational studies that reported assessments of cardiovascular outcomes and death of participants using angiotensin-converting enzyme inhibitors, angiotensin receptor blockers, beta-blockers, mineralocorticoid-receptor antagonists and Ivabradine, were included. The primary outcome was total mortality. Secondary outcomes included changes in left ventricular ejection fraction (LVEF), serum natriuretic peptide levels (BNP), and heart rate (HR). Data were extracted for eligibility by two independent reviewers. Random-effects meta-analysis was used to pool results.

**Results:** Twelve studies with 439 patients were included in our meta-analysis. Treated patients have lower HR, mean difference of −17 beats per minute (CI [−25]–[−9], *p* < 0.01). The LVEF was improved in treated patients, with a mean difference of LVEF of 3.77% (CI 0.44–7.12, *p* < 0.03). Although mortality rates did not reach statistical significance there was a trend for total mortality reduction (hazard ratio 0.36, CI (0.1–1.25), *p* = 0.107) and for BNP reduction (SSMD: 0.141, CI ([−0.19]–[0.47]), *p* = 0.3).

**Conclusion:** Pharmacologic treatment for DMDCM patients is associated with decreased HR and improved LVEF. Therefore, DMDCM patients may benefit from implementing guideline therapy for HF.

**Clinical Trail Registration**: PROSPERO, identifier [CRD42018111888].

## Introduction

DMD is the most common and severe disease in muscular dystrophies, an inherited neuromuscular disorder characterized by severe clinical symptoms such as muscle weakness, severe disability, and rapid progression. DMD affects nearly 16–20 out of 100,000 live births, is a recessive hereditary X-linked condition, thus mainly affecting males. However, women exhibit a milder phenotype of the disease (McNally et al., 2015; Romitti et al., 2015).

Numerous mutations in the dystrophin gene cause DMD. These may be deletions or duplication of DNA sequences (70–80% of the cases) or point mutations in some instances. The mutated dystrophin gene leads to critical protein deficiency, which in turn progresses into muscle fiber damage (Muntoni et al., 2005; Aartsma-Rus et al., 2006; Ryder et al., 2017).

Lack of cardiac dystrophin protein may damage calcium channel functionality which is critical for muscle contractions. As a result, intracellular calcium levels increase, leading to proteases activation, protein destruction, fibrosis, and eventually cell death, (Whitehead et al., 2006; Falzarano et al., 2015) which may lead to DMDCM in young and adolescent patients up to their 30s (Law et al., 2020). HF is one of the primary causes of morbidity and mortality in DMD patients, (Bushby et al., 2003; Verhaert et al., 2011; McNally et al., 2015)^,^ and the pathophysiology of DMDCM is related to sub-pericardial fibrosis of the myocardium (Danialou et al., 2001).

DMDCM is typically asymptomatic for extended periods. Thus early detection is challenging, (Takenaka et al., 1993; Sasaki et al., 1998; Bushby et al., 2003; Giglio et al., 2003; American Academy of Pediatrics Section on Cardiology and Cardiac Surgery, 2005) nevertheless early diagnosis is generally crucial to mitigate HF symptoms, myocardial damage and begin anti-remodeling cardiac therapy with various medications, including Beta Blockers and Renin-Angiotensin-Aldosterone system antagonists. ACEi or ARBs are used as first-line treatment in DMDCM, and BBs are added later to the treatment (Viollet et al., 2012; D’Amario et al., 2017; Birnkrant et al., 2018).

While almost all DMD patients that reach their 30s develop cardiomyopathy., DMD is a rare disease, and the course of HF progression and the impact of therapy on the cardiomyopathy in these patients lack specific guidelines leading to suboptimal treatment.

This meta-analysis aims to study the efficacy and safety of pharmacological treatment for DMDCM.

## Methods

We performed a systematic review and a meta-analysis to assess HF therapy efficacy in patients with DMD.

The research was conducted following the PRISMA extension statement for meta-analyses. Random-effects meta-analysis was used to pool analyses (see Supplemental Material, Supplementary Table S1). (Moher et al., 2015) A review of observational studies is reported following the MOOSE protocol (see Supplementary Table S2). (Stroup et al., 2000) The study protocol was registered in PROSPERO (registry number: CRD42018111888, date: October 2018). No approval from an institutional review board was required.

### Data Sources and Searches

PubMed (MEDLINE), Embase, and the Cochrane library were searched through October 2018; the search was updated through January 2021. RCTs, case-control studies, and observational studies that reported assessments of cardiovascular outcomes and death of participants using ACEi, ARBs, BBs, MCRAs, and Ivabradine were included without language or date restrictions. No data were available regarding Sacubitril/valsartan regimen and thus was excluded from this analysis.

The following MeSH, Emtree, and search keywords were used to identify the relevant studies: Duchenne Muscular Dystrophy, HF, ACEi, angiotensin-converting enzyme inhibitor, ARB, angiotensin receptor blocker, ARNI, angiotensin receptor, Neprilysin inhibitor, Beta-blocker, β- blockers, MR antagonist, Mineralocorticoid receptor antagonist, Spironolactone, Eplerenone, Anti-mineralocorticoid, Ivabradine, Digoxin, Cardioprotective therapies.

The precise search query that was used is described in the Supplemental Material.

### Study Selection, Data Extraction, and Outcomes

Two independent reviewers (B.H.R and B.B) screened the search results using the Rayyan QCRI web application for systematic reviews (Ouzzani et al., 2016). Any disagreements resolved by consensus were referred to a third reviewer (I.M).

Initially, studies underwent selection according to titles and abstracts, and a second selection phase was performed by thoroughly reading the articles. Some exceptions were made whenever a full version was unavailable (Duboc et al., 2005), but data were available in a conference abstract.

A description of the characteristics of the included studies can be found in Table 1.

**TABLE 1:** Characteristics of studies included in analysis.

Study	Study design	Length of follow up	n	Mean age (years)	Heart function at baseline	Treatment group	Comperator group	Study endpoints	Conclusion
Duboc et al. (2005)	Phase I- RCT.	6 years	57	9.5–13	normal cardiac examinations and function, LVEF>55%	*n* = 28 patient on Peridopril	*n* = 29 patients on placebo	mortality	trend towards lower mortality after 6 years of peridopril treatment in DMD patients, and good tolerance
	Phase II- Cohort Study								
Jefferies et al. (2005)	Cohort Study	3.3 years	**31**	15.4	After the first abnormal echocardiogram- ACEi or BB therapy was started	*n* = 18 patients ACEi + BB.	*n* = 13 patients ACEi only	LVEF%	early diagnosis and treatment of dilated cardiomyopathy can lead to ventricular remodeling
Kajimoto et al. (2006)	Cohort Study	2–3 years	28	17 ± 5	LVEF<55%	*n* = 13, ACEI + BB	*n* = 15, ACEi only	HR, BNP	Carvedilol plus an ACEI improves left ventricular systolic function
Rhodes et al. (2008)	Cohort Study	6 months	22	21.5 ± 8.4	LVEF<50%	*n* = 22 BB	BB before and after treatment	HR, LVEF%	carvedilol therapy can be initiated and appears to be well tolerated
Matsumura et al. (2010)	Cohort Study	5 years	54	BB group: 23.2 ± 8.5	All patients with an EF< 50% received ACEi	*n* = 41 BB group	*n* = 13	mortality	carvedilol is relatively safe and can prevent cardiac events
				Non BB group: 19.3 ± 4.7			non BB		
Viollet et al. (2012)	Cohort Study	48 months	42	14.8 ± 4.6	LVEF<55%	n-24. ACEi (lisinopril)+BB.	*n* = 30. ACEi only (lisinopril)	LVEF%	treatment with ACEi with or without BB can delay progression of cardiomyopathy
Raman et al. (2015)	RCT	12 months	42	14.5	LVEF>45%	*n* = 20, eplerenone	*n* = 22 placebo	Mortality	addition of eplerenone to background ACEI or ARB therapy attenuates the progressive decline in left ventricular systolic function
Silva et al. (2017)	RCT	2 years	42	12.1 ± 2.7	LVEF>50%	*n* = 21 ACEi. no dysfunction- treatment	*n* = 21	LVEF%, mortality	the use of ACEi slows myocardial fibrosis progression at a 2-year follow-up
							no ACEi		
							no dysfunction		
Dittrich et al. (2019)	RCT	3.5 years	41	10–14	LV-FS ≥ 30%	*n* = 21 ACEi (enelapril) +BB (metoprolol)	*n* = 17, placebo	HR, BNP	Enalapril and metoprolol treatment is suggestive to delay the progression of the intrinsic cardiomyopathy to left ventricular failure, but did not reach statistical significance, probably due to insufficient sample size
Adorisio et al. (2019)	Cohort Study	4.5 years	20	15.0 ± 3.5	LVEF<40%. Chronic HF treatment with an ACEi inhibitor during the study period	*n* = 9 BB + IVABRADINE	*n* = 11 BB onlu	HR, LVEF%	HR reduction strategy, seemed to be effective in reducing the incidence of acute adverse events
Aikawa et al. (2019)	Cohort Study	median of 3 years	21	10.1	5 were started on ACEI at LVEF ≥55% and 10 at LVEF <55%	*n* = 21 ACEi	ACEi before and after treatment	LVEF and the extent of myocardial late gadolinium enhancement	ACEI attenuated the age-related decline in LVEF only in patients with reduced LVEF. However, ACEI use did not affect the age-related increase in myocardial fibrosis
Kim et al. (2020)	Case-control Study	mean of 1.57 years	48	15.35	Group 1 59.297 ± 8.407, Group 2 33.923 ± 11.547	LV EF ≥ 45% (*N* = 30)	before and after treatment	IVSs, LVIDs,LVPWd, LVEF,FS, DT slope	For patients with lower LVEF, ACEi might be beneficial to preserve cardiac function
						LV EF < 45% (*N* = 18)			

n, number of subjects; RCT, randomized, double-blind, placebo-controlled trial.; LVEF, left ventricular ejection fraction; DMD, duchenne muscular dystrophy; ACEi, Angiotensin-converting-enzyme inhibitors; BB, beta blockers; HR, heart rate; BNP, brain natriuretic peptide; LV-FS, left ventricular fractional shortening; IVSs, Interventricular septal thickness at end systole; LVIDs, Left ventricular internal diameter end systole; LVIDs, Left ventricular internal diameter end systole; LVPWd, Left ventricular posterior wall thickness end diastole; FS, fractional shortening; DT,deceleration time

### Statistical Analysis

Randomized controlled studies were evaluated by the Cochrane Collaboration’s Risk of Bias Tool, (Higgins et al., 2011)^,^ which is used to assess bias risk (high, low, or unclear) in the following aspects: selection bias, performance bias, detection bias, reporting bias, and attrition bias. The NOS scale was used for assessing the risk of bias and the quality of nonrandomized studies (Wells et al., 2010). The scale is based on eight criteria and provides a star rating score ranging from 0 (high risk for bias) to 9 (low risk for bias). The evaluation summary was performed for every outcome. In each study, the bias was assessed by two independent reviewers, with disagreements being resolved by reaching an agreement or contacting a third reviewer.

Publication bias was assessed by visual inspection of the funnel plot and by the Egger test.

Data from all arms of the multi-arm experiments were retrieved. Dichotomous data were measured as the number of events in the intervention groups and the control group and participants. Continuous data were evaluated as mean change from baseline and standard error when available. When the results were reported before and after measurement, we used these results, linking them to the mean difference using the correlation estimated from studies with complete information. In two studies, the Hidemi et al. and Adorisio, a separate analysis was performed for patients treated with one therapy as compared to two medications therapy (ACEi vs. ACEi + BB, BB vs. BB + ivabradine, respectively) (Kajimoto et al., 2006; Adorisio et al., 2019).

For ethical reasons, in most studies, HF treatment for DMD patients assesses medication combinations as opposed to a single medication. Therefore, our analysis evaluated the pooled effect of multiple medication treatments, as were reported.

The data were analyzed using the Comprehensive Meta-Analysis software.

It is reasonable to assume that each study has unique characteristics that may affect the effect size, implying sampling variability. Therefore, a random-effects model was used due to the assumption that the effect size varies across studies because of substantial differences between the interventions’ effect and sampling variability (Riley et al., 2011).

The primary outcome in all studies was mortality, which was measured as the number of events per group. We used the random-effects model to calculate the pooled odds ratio for dichotomous data and the 95% CI. Secondary outcomes were: change in ejection fraction, brain natriuretic peptide, and heart rate. These results were measured by calculating the mean quantitative difference before and after medication. Moreover, in the case-control study, the difference between the treatment and control groups was measured.

### Trial Sequential Analysis

The following may lead to spurious *p*-values: bias from trials with low methodological quality, outcome measure bias, publication bias, early stopping for a benefit, and small trial bias. Existing literature is sparse. Therefore a meta-analysis may incur systematic errors (bias) or random errors (play of chance) in the process of concluding.

To examine the strengths of meta-analysis evidence, we performed a trial sequence analysis with the TSA software (Thorlund et al., 2011). TSA was performed for any pooled analysis that did not reach statistical significance, and information size was calculated to assess if sufficient cumulative numbers of subjects were present in the studies included in our analysis. Trial sequential boundaries adjust the CIs and reduce type I errors. When the cumulative z-curve crosses the trial sequential monitoring boundary, a sufficient level of evidence for the anticipated intervention effect may have been reached, and no further trials are needed, and vice versa.

The information size is defined as the number of events and participants necessary to reject or detect an a priori assumed intervention effect in a meta-analysis (Wetterslev et al., 2009). The required information size was calculated based on the proportion of patients with an outcome in the control and intervention groups, a relative risk reduction of 67.27%. We appropriately adjusted the TSAs for heterogeneity (diversity adjustment) according to an overall type I error of 5% and a power of 80%.

## Results

### Literature Search

A systematic search yielded 528 citations. Preliminary screening excluded 109 duplicate citations. The 419 remaining titles were reviewed by the abstract. A total of 341 citations were excluded, leaving 78 records for full-text review. According to inclusion criteria, the full review excluded 66 additional citations, leaving twelve records for analysis (Supplementary Figure S1).

### Study Characteristics

Twelve studies were included in our meta-analysis (Duboc et al., 2005; Jefferies et al., 2005; Kajimoto et al., 2006; Rhodes et al., 2008; Matsumura et al., 2010; Viollet et al., 2012; Raman et al., 2015; Silva et al., 2017; Aikawa et al., 2019; Adorisio et al., 2019; Dittrich et al., 2019; Kim et al., 2020). Four studies were RCTs, (Duboc et al., 2005; Raman et al., 2015; Silva et al., 2017; Dittrich et al., 2019) seven were observational, (Jefferies et al., 2005; Kajimoto et al., 2006; Rhodes et al., 2008; Viollet et al., 2012; Adorisio et al., 2019; Aikawa et al., 2019; Kim et al., 2020)and one study was open-label. Study characteristics are summarized in Table 1 (Matsumura et al., 2010).

In total, 439 patients were included in the meta-analysis, of which 286 DMD patients were treated for HF (BBs, ACEi, Eplerenone, Ivabradine, and combination, as described in Table 1).

In five of the twelve studies, (Duboc et al., 2005; Matsumura et al., 2010; Raman et al., 2015; Silva et al., 2017; Dittrich et al., 2019) the control was placebo in addition to supportive care. Moreover, the other seven studies examined the effect of HF medications on patients before and after treatment.

From the ACEi class, the tested medications were: Enalapril, (Jefferies et al., 2005; Kajimoto et al., 2006; Silva et al., 2017; Aikawa et al., 2019; Dittrich et al., 2019; Kim et al., 2020) Perindopril, (Duboc et al., 2005) Lisinopril, (Jefferies et al., 2005; Viollet et al., 2012) Captopril, (Jefferies et al., 2005) and Cilazapril (Kajimoto et al., 2006; Aikawa et al., 2019; Kim et al., 2020).

Of the BB family, Carvedilol was mainly tested, (Jefferies et al., 2005; Kajimoto et al., 2006; Rhodes et al., 2008; Matsumura et al., 2010; Adorisio et al., 2019) but some studies also examined Metoprolol’s administration (Jefferies et al., 2005; Viollet et al., 2012; Dittrich et al., 2019). Other pharmacological treatments for HF given to patients were Ivabradine and Eplerenone (Raman et al., 2015; Adorisio et al., 2019).

### Outcomes

#### Heart Rate Reduction

**FIGURE 1:**
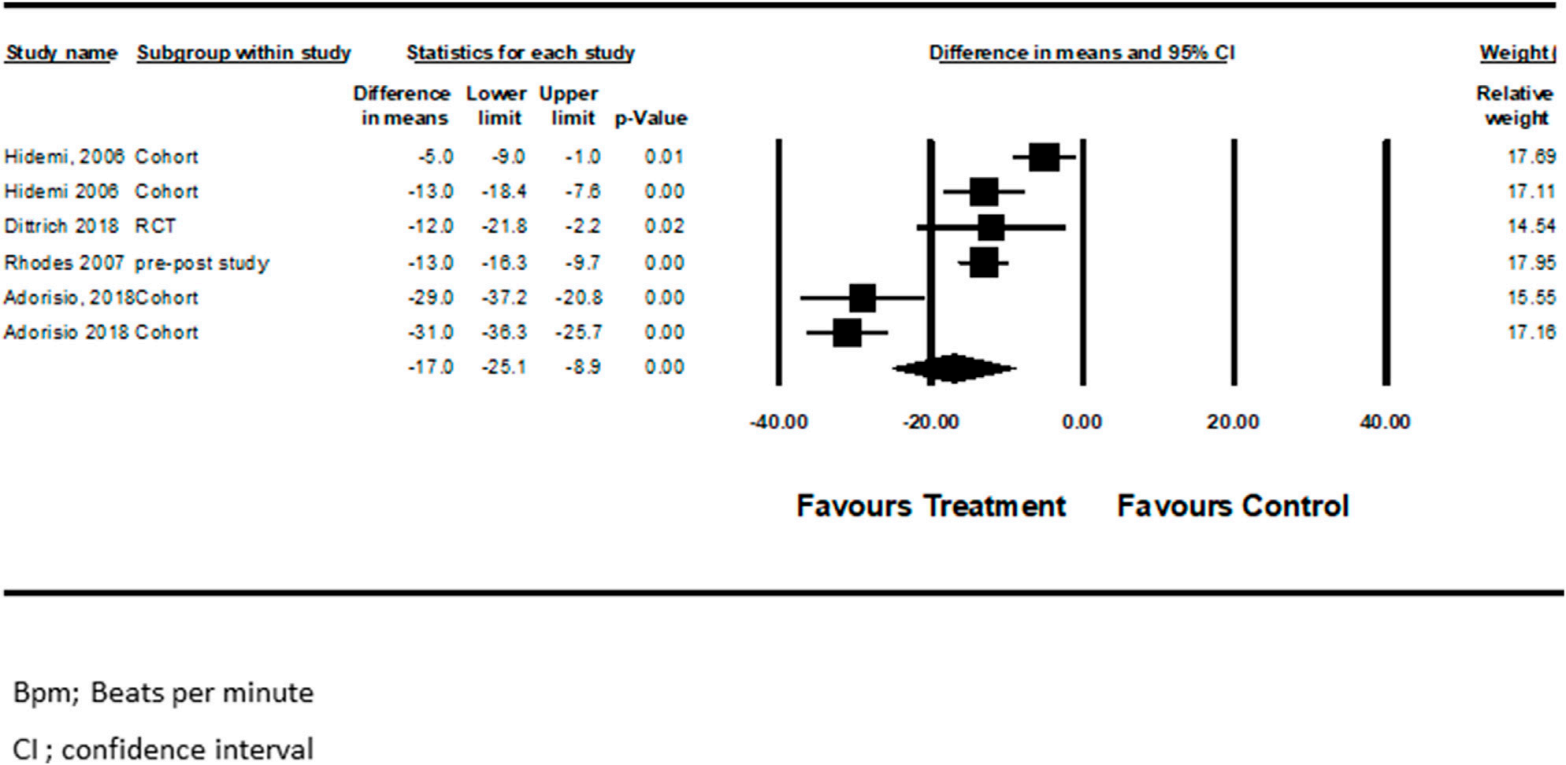
The actual differences in bpm following HF treatment versus control.

Four studies examined the effect of pharmacotherapy on HR among participants (Kajimoto et al., 2006; Rhodes et al., 2008; Adorisio et al., 2019; Dittrich et al., 2019). The pooled analysis showed that pharmacological treatment for HF in these patients was associated with lower HR (mean difference = −17.02 beats per minute (bpm), CI ([−25.1]–[−8.9]), *p* < 0.0004, I^2^ = 93%) (Figure 1), suggesting a beneficial effect of therapy for HF on DMD patients compared with those who did not receive treatment or those who did not receive combination therapy.

### Change in LVEF %

**FIGURE 2:**
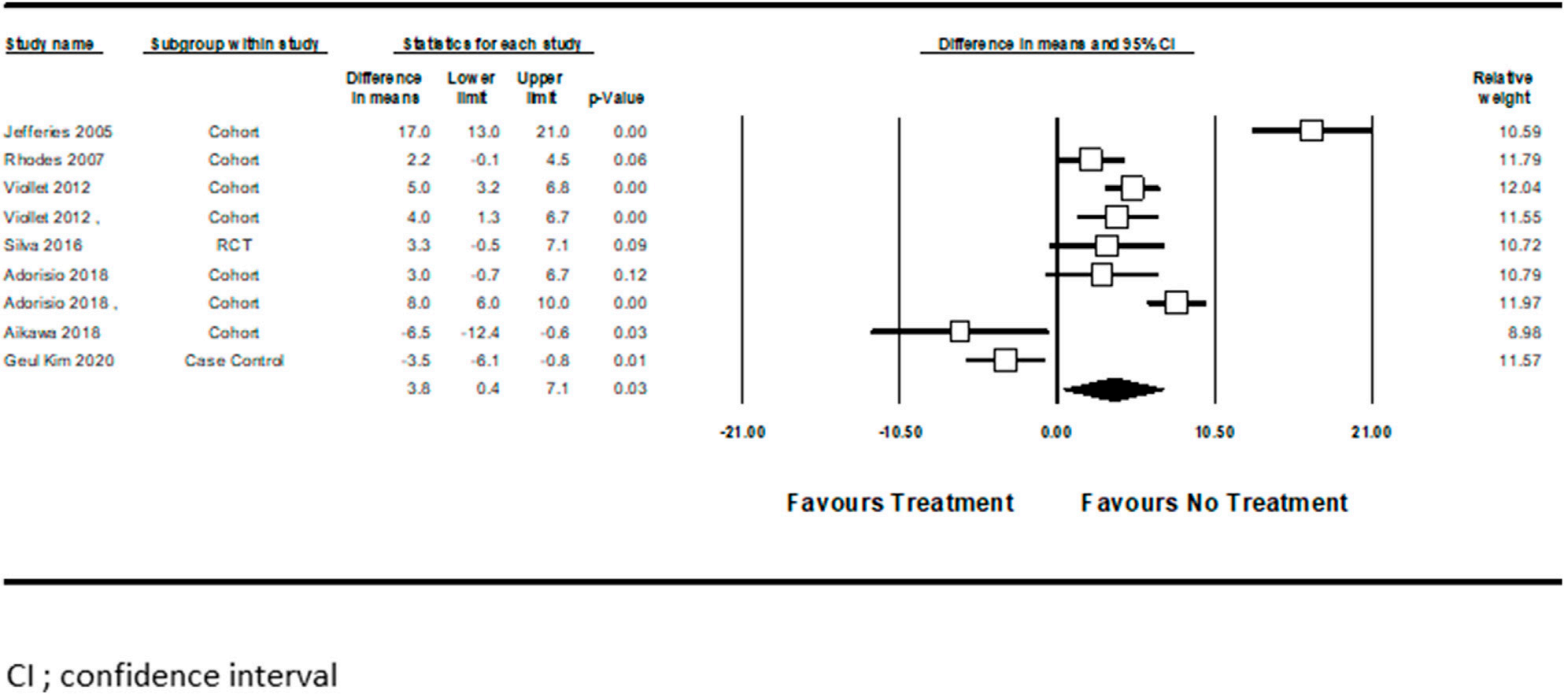
The differences in means for LVEF (%) following HF treatment versus control.

Seven studies examined pharmacotherapy’s effect on LVEF% changes from baseline (Jefferies et al., 2005; Rhodes et al., 2008; Viollet et al., 2012; Silva et al., 2017; Adorisio et al., 2019; Aikawa et al., 2019; Kim et al., 2020). Viollet et al. (2012) performed separate analyses for those who received only ACEi and those who received a combination of ACEi + BBs. In another study, Adorisio et al. (2019) compared patients receiving BBs vs. those receiving a combination of BBs + Ivabradine. Pooled analysis from the above studies showed that drugs for HF were associated with improved LVEF in the treated patients (mean difference = 3.8, CI (0.4–7.1), *p* < 0.03, I^2^ = 92%). (Figure 2).

### Serum BNP Levels (pg/ml)

**FIGURE 3:**
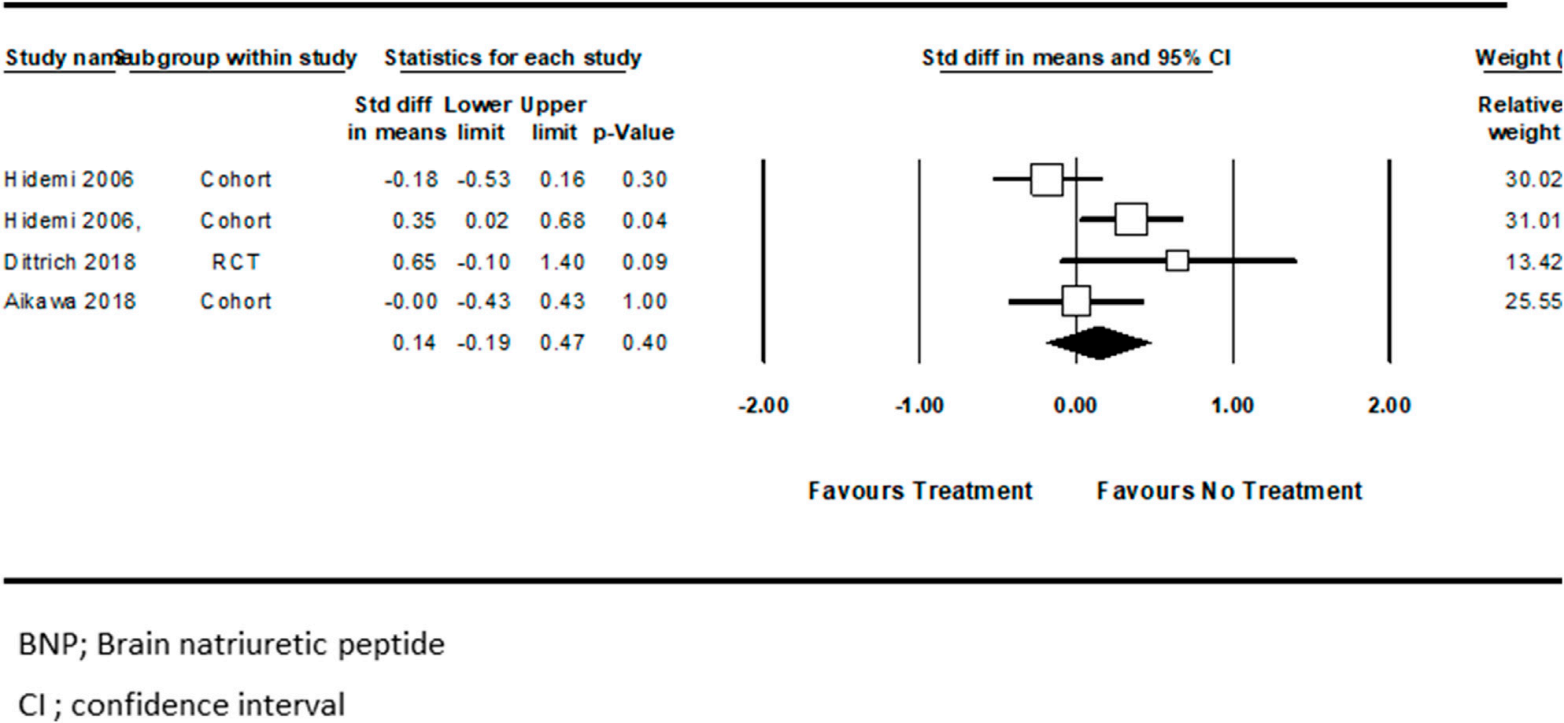
The standard differences in means for BNP (pg/ml) following HF treatment versus control.

Three studies examined pharmacotherapy’s effect on serum BNP level changes from baseline (Kajimoto et al., 2006; Aikawa et al., 2019; Dittrich et al., 2019). Hidemi et al. (Kajimoto et al., 2006) performed the analysis separately for ACEi treated patients compared with those who received a combination of ACEi + BBs. Pooled analysis showed no significant difference associated with BNP level reduction between these two study groups (standard difference in mean = 0.14, CI ([−0.19]–[0.47]), *p* = 0.4, I^2^ = 58%) (Figure 3).

### Mortality

**FIGURE 4:**
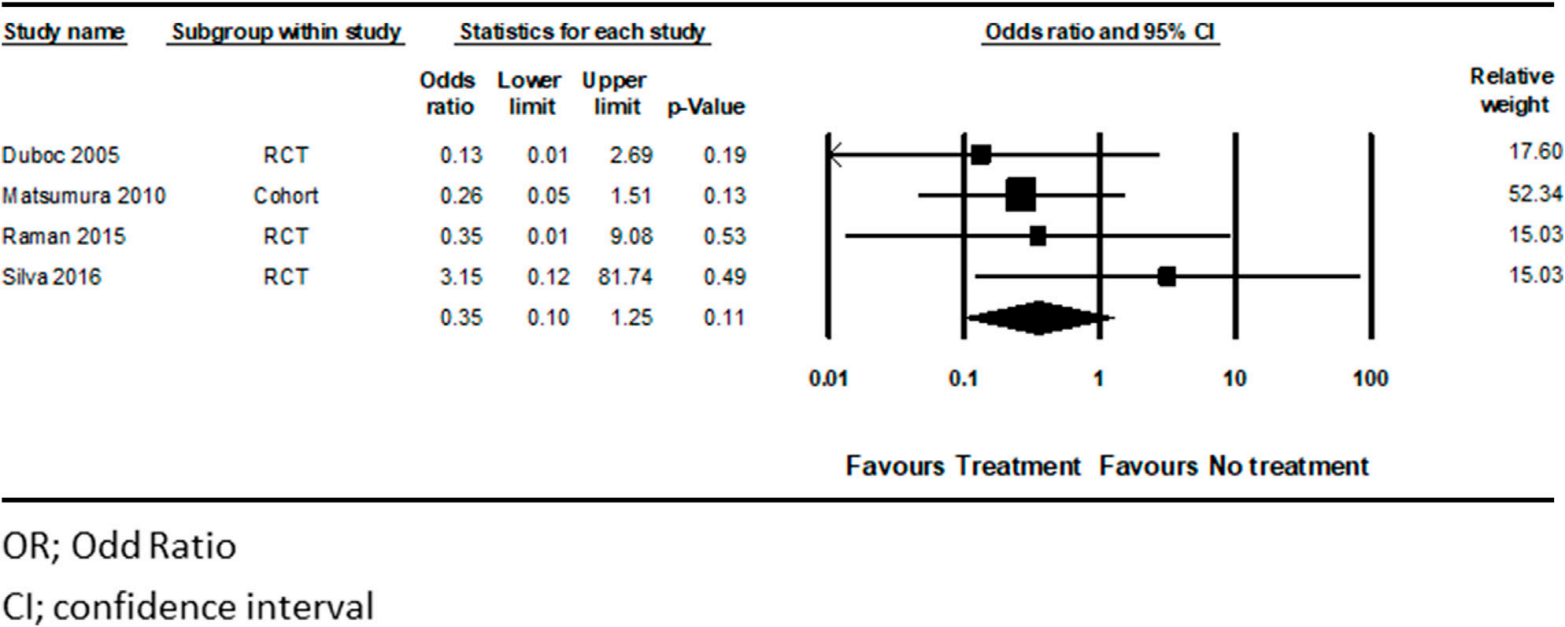
The Odd Ratio for mortality following HF treatment versus control.

Four studies totaling 195 patients, (Duboc et al., 2005; Matsumura et al., 2010; Raman et al., 2015; Silva et al., 2017) examined HF treatment on mortality in DMD patients. There was a decreased risk for mortality in the treated patients, compared to untreated DMD patients, as demonstrated by the pooled analysis; pooled OR for the treatment group was 0.36 CI (0.10–1.25), *p* < 0.11, I^2^ = 0%), compared with the untreated group (Figure 4). However, this outcome did not reach statistical significance.

### Sensitivity Analysis

We performed a sensitivity analysis to include only studies with a lower risk of bias, demonstrated by the Cochrane Collaboration’s Risk of Bias Tool (Higgins et al., 2011). We determined that a selection bias may be present in Silva et al. and thus excluded from the sensitivity analysis (Silva et al., 2017). Without Silva et al., we were able to show that HF treatment for DMD patients was associated with a statistically significant reduction in mortality (pooled OR 0.24, CI (0.06–0.95), *p* < 0.04) (Supplementary Figure S2).

### Trial Sequential Analysis for Mortality Outcome

According to TSA, the number of mortality events that was calculated as a threshold for statistical significance power was 1,024, while the actual number of patients that died in all the studies included in our meta-analysis was lower, suggesting that there is no statistical power to determine whether or not an association exists between the pharmacological treatment and the risk for mortality (Supplementary Figure S3). Moreover, we did not see a trend towards an effect in the TSA.

### Risk of Bias

The overall ROB among the included studies was low. All the studies included in our meta-analysis had a small sample size. The ROB assessment is summarized in Supplementary Figures S4, S5. Due to less than ten studies included in the meta-analysis, we cannot test for funnel plot asymmetry to assess publication bias.

## Discussion

To our knowledge, this is the largest meta-analysis performed to examine the effectiveness of anti-remodeling cardiac therapy for DMDCM patients. The pooled results from our meta-analysis indicate that anti-remodeling cardiac therapy has beneficial effects on DMDCM patients, associated with decreased heart rate and increased LVEF.

The use of anti-remodeling cardiac therapy for DMDCM patients was investigated previously in the Cochrane review, the results of the analysis were inconclusive. Nevertheless, our finding also supports the conclusion of the Cochrane review that early treatment with ACE inhibitors or ARBs may be comparably beneficial for people with dystrophinopaty. However, Cochrane meta-analysis included only two RCT of ACE inhibitors treatment (one of them compared losartan with lisinopril without placebo control), without beta-blockers, and examined only EF outcome (Bourke et al., 2018)

We also examined the decreased risk for mortality, which did not reach statistical difference in treated DMDCM patients for anti-remodeling cardiac therapy, compared with nontreated patients. The lack of association may be due to a minimal number of death events in each research arm. Due to a small number of studies, we performed a sensitivity analysis excluding data from Silva et al. (2017) ^,^ which did not report any deaths in the control group, due to possible biases present in this study. In this sensitivity analysis, we showed an association with a decreased risk of mortality. A larger sample size might have sufficient statistical power to show a statistically significant association.

Early detection of DMDCM has become relevant since the introduction of anti-remodeling cardiac therapy may slow adverse effects and attenuate HF symptoms (Nigro et al., 1990). Moreover, several studies demonstrated that early initiation of treatment, before the onset of symptoms and prior to LVEF, yields better outcomes (Duboc et al., 2005; Ogata et al., 2009). Currently, the treatment of choice for HF includes using ACEi as a first-line treatment and the addition of BBs (Ponikowski et al., 2016; Yancy et al., 2017). Our analysis results support this approach and show an improvement in HF parameters using these medication classes.

DMDCM is a common complication among DMD patients. Negro et al. examined the incidence of cardiac disease in 328 DMD patients. Preclinical cardiac involvement was found in 25% of patients under six years old, increasing to 59% between ages 6–10 years and then declining in incidence with age. Clinically apparent DMDCM is first evident at ten years, increasing incidence with age up to being present in all patients over 18 years. (Nigro et al., 1990).

Anti-remodeling cardiac therapy in DMDCM relies on evidence acquired from other HF populations, using standard HF strategies and remaining suboptimal (Bushby et al., 2003).

Spurney et al. reported in their DMDCM study of 340 patients that 57% (27 of 47) of patients were not taking any therapy. In a different study, therapy was used on 12% (15 of 127) of DMD patients without cardiomyopathy (Spurney et al., 2014).

An increase in all-cause mortality by 14% at every ten bpm HR increase was demonstrated in the general population (Nigro et al., 1990). In HF patients resting HR > 80 bpm could cause myocardial dysfunction, further deteriorating HF. It is well known that the cornerstone medications for chronic systolic HF are ACEi and BBs, which reduce HR (Ponikowski et al., 2016; Yancy et al., 2017). Our results also support this concept in DMDCM, as the statistically significant HR decrease, was observed in all studies, not only in those examining medications from the BB family (Kajimoto et al., 2006; Rhodes et al., 2008; Adorisio et al., 2019; Dittrich et al., 2019).

As described above, four studies with 195 patients were included in our analysis for mortality outcome, which did not reach statistical significance, corroborated by the results of our TSA. Moreover, larger studies, CONSENSUS,-1, and SOLVD, evaluated the effect of Enalapril on HF patients and reported a decreased risk of mortality. The CONSENSUS-1 trial included 253 participants reporting a 40% mortality reduction at six months in patients with severe HF treated with Enalapril in addition to standard therapy (The Consensus Trial Study Group, 1987; The SOLVD Investigators, 1991). Similarly, the SOLVD study included 2,569 participants of less symptomatic patients with dilated cardiomyopathy, reporting a 16% mortality reduction with Enalapril (The SOLVD Investigators, 1991). These studies include a larger number of patients compared to our analysis of DMD patients; therefore, the sample size may be the reason why our results are not statistically significant.

BNP is a neuro-hormone secreted by the heart chamber muscle cells due to increased surface tension, pressure, and volume. When active, BNP causes salt and water secretion (natriuresis), vasodilation, inhibition of the renin-angiotensin system, and the adrenergic system. BNP levels rise in patients with HF and have prognostic significance (Mohyuddin et al., 2005; Law et al., 2020). Our results did not show a reduction in BNP levels, possibly for several reasons. Only two studies examined the effect of HF treatment on BNP levels in DMD patients. Thus BNP may not be an indicative marker. Mohyuddi et al. reported normal BNP levels in a majority of DMD patients with LV dysfunction. Moreover, BNP is mildly elevated when LVEF is markedly reduced (Mohyuddin et al., 2005).

While there is universal agreement that steroids prolong ambulation and improve respiratory muscle strength. In the DMD patient, the cardioprotective effect of steroids is controversial (Silversides et al., 2003; Markham et al., 2005; Schram et al., 2013). However, it has been reported that patients with DMD treated with corticosteroids have a 20% decrease in the probability of developing Duchene cardiomyopathy compared with untreated patients (Barber et al., 2013). A retrospective study found that the progressive decline in cardiac function of patients with Duchene cardiomyopathy could be altered by steroid treatment (Markham et al., 2005). Schram et al. reported that in patients with DMD, steroid therapy was associated with noticeably lower cause mortality, due mainly to a significant reduction in heart failure-related deaths (Schram et al., 2013). Furthermore, deflazacort treatment in DMD patients has been associated with preserving cardiac function (Silversides et al., 2003).

Novel Echocardiography or MRI capabilities, such as circumferential strain and Late Gadolinium Enhancement, for detecting cardiac fibrosis correlated with cardiac outcomes (Hor et al., 2013; Amedro et al., 2019; Yu et al., 2019). Imaging may also allow pre-symptomatic and pre-phenotypic identification of cardiac involvement, encouraging early treatment that might lead to better outcomes. Future meta-analyses may incorporate these modalities as outcomes.

Study limitations: First, this meta-analysis is based on a few studies with a small number of patients. Each of the studies included patients with different cardiac function at baseline and different mean age, which might have contributed to the heterogeneity in our pooled analysis. However, we used a random-effect model and sensitivity analysis to evaluate the outcomes. Second, Some patients included in these studies were not DMD but were diagnosed with Baker muscular dystrophy, for instance. However, the number of these patients was very low (14 out of 195). Third, In recent years the management of non-DMD-related HFrEF experienced a surge of novel drug candidates. Drugs classes such as the Angiotensin Receptor-Neprilysin inhibitors (ARNi) and Sodium-Glucose co-Transporter-2 inhibitors (SGLT2i) demonstrated meaningfully improved outcomes, including survival and reduced HF hospitalizations (McMurray et al., 2014; Lee et al., 2019; Packer et al., 2020).Soluble guanylate-cyclase inhibitors and myosin activators led to significant, yet less impressive results (Armstrong et al., 2020; Teerlink et al., 2021). It is of great interest to test the potential effects of such drugs on DMDCM, especially SGLT2i and ARNi, as their use results in attenuated cardiac fibrosis (Lee et al., 2019). Another interesting class is the non-steroidal mineralocorticoid receptor agonists, which might exert similar effects as the steroidal counterparts with the advent of fewer hormone-related side effects (Pei et al., 2018). Cooperation of the pharmaceutical companies, physicians, and families is needed for carefully testing these options in DMDCM. Forth, our analysis included common HF medication. We did not include oral corticosteroids in this analysis. Further meta-analysis is required to evaluate the cardioprotective effect in DMD patients (McMurray et al., 2014).

## Conclusion

Pharmacologic treatment for DMDCM patients is associated with decreased HR and improved LVEF. Therefore, DMDCM patients may benefit from implementing guideline therapy for HF. The time and age of treatments should further be studied, and additional new treatment options for DMD patients should be monitor.

## Data Availability

The original contributions presented in the study are included in the article/Supplementary Material, further inquiries can be directed to the corresponding author.

## Abbreviations

ACEi, Angiotensin-Converting Enzyme inhibitors; ARBs, Angiotensin Receptor Blockers; BBs, Beta-Blockers; BNP, Brain Natriuretic Peptide; CI, Confidence Interval; DMD, Duchenne Muscular Dystrophy; DMDCM, Duchenne Muscular Dystrophy Cardiomyopathy; HF, Heart Failure; HR, Heart Rate; LVEF, Left Ventricular Ejection Fraction; MCRAs, MineraloCorticoid-Receptor Antagonists; MOOSE, Meta-analysis Of Observational Studies in Epidemiology; NOS, Newcastle-Ottawa Scale; PRISMA, Preferred Reporting Items for Systematic Reviews and Meta-Analyses; RCTs, Randomized Controlled Trials; ROB, Risk Of Bias; TSA, Trial Sequential Analysis.
